# A co-culture system of rat synovial stem cells and meniscus cells promotes cell proliferation and differentiation as compared to mono-culture

**DOI:** 10.1038/s41598-018-25709-w

**Published:** 2018-05-16

**Authors:** Xing Xie, Jingxian Zhu, Xiaoqing Hu, Linghui Dai, Xin Fu, Jiying Zhang, Xiaoning Duan, Yingfang Ao

**Affiliations:** Institute of Sports Medicine, Beijing Key Laboratory of Sports Injury, Third Hospital of Peking University, Beijing, China

## Abstract

A meniscus tear often happens during active sports. It needs to be repaired or replaced surgically to avoid further damage to the articular cartilage. To address the shortage of autologous meniscal cells, we designed a co-culture system of synovial stem cells (SMSCs) and meniscal cells (MCs) to produce a large cell number and to maintain characteristics of MCs. Different ratios of SMSCs and MCs at 3:1, 1:1, and 1:3 were tested. Mono-culture of SMSCs or MCs served as control groups. Proliferation and differentiation abilities were compared. The expression of extracellular matrix (ECM) genes in MCs was assessed using an ECM array to reveal the mechanism at the gene level. The co-culture system of SMSCs/MCs at the ratio of 1:3 showed better results than the control groups or those at other ratios. This co-culture system may be a promising strategy for meniscus repair with tissue engineering.

## Introduction

Meniscal tear is a common knee injury and often requires surgical repair or replacement to avoid further damage to the articular cartilage. Currently, tissue engineering is a promising solution to promote meniscal healing, where a critical element is the suitable cell source. Because of the lack of autologous meniscal cells, attempts are being made to find alternative solutions for meniscal tissue engineering. Stem cells from various tissues including bone, cartilage, muscle, and nerves have been used in tissue engineering^1,2^. The challenge for meniscus regeneration is that meniscal cells (MCs) are scarce and heterogeneous. At least two types of cells coexist in different zones of the meniscus, including round chondrocyte-like cells and spindle-shaped fibroblast-like cells. Therefore, it is difficult to induce stem cells to differentiate into MCs *in vitro*. Autologous MCs, though capable of generating meniscus-like tissues, have limited applications because of the insufficient number of cells and poor capacity for proliferation.

Recently, co-culture system was designed to mix two or more types of cells in one culture system. Different cells influence each other via direct contact and the indirect cytokine paracrine route^3,4^. Both cellular growth and differentiation ability in the co-culture system can be different from those in a mono-culture^5^. Several studies have been conducted using the co-culture theory for cartilage repair. In these studies, MSCs mediated a stimulatory effect on chondrocytes and up regulated the cartilaginous matrix formation of chondrocytes^6^. This stimulatory effect, defined as the trophic effect of MSCs^2,7–10^, is believed to be important for co-culture system. It was reported that synovium-derived MSCs play a vitally important role in the natural repair procedure of a meniscal tear^11^. These cells take part in the inflammation process and tissue reconstruction in the meniscus. Some studies have shown that native SMSCs are likely to be suitable cell sources for repair of meniscus lesions in tissue engineering^12–15^. Therefore, we used SMSCs and MCs together as cell sources, and different ratios of the two kinds of cells were tested in the co-culture system. Cui X. *et al*. found that the co-culture of MCs and MSCs at the ratio of 75:25 yielded the highest production of type I collagen and glycosaminoglycan (GAG) as well as the lowest expression of hypertrophic genes, such as *col10a1* and *mmp3*. Though all co-culture conditions showed better meniscus extracellular matrix (ECM) production and hypertrophic inhibition as compared to MSC culture alone^16^. However, in this study, no data on ECM were obtained as compared to MC culture alone. Another study used the ratio of 50:50 for SMSCs/MCs on a small-intestine submucosa for meniscal tissue engineering^17^. The co-culture group showed higher expression of chondrogenic genes. However, the optimal proportion of co-cultured cells still remains unknown. To determine the best ratio of SMSCs to MCs, we designed a co-culture system at different ratios and compared the cell viability and differentiation after co-culture for 14 days. The expression of ECM genes in MCs was also compared between the groups of different ratios and the MCs culture alone, to understand the mechanism of cell interaction in the co-culture system. The optimal SMSCs/MCs ratio was determined based on these results.

## Results

### Primary cell cultures and characterisation

**Figure 1:**
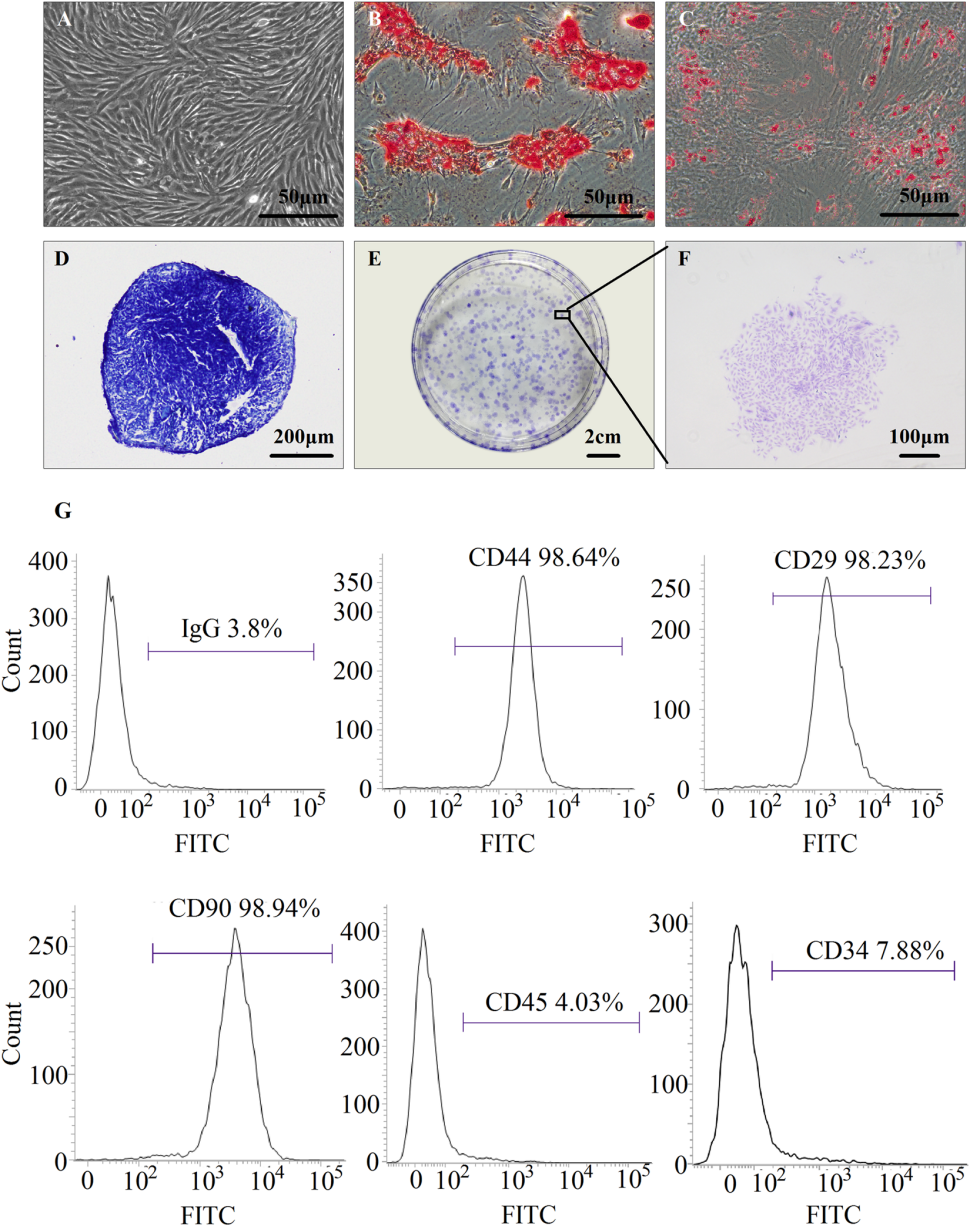
The characteristics of rat SMSCs were examined by immunohistochemistry, a colony formation assay, and flow cytometric analysis. The cells at passage 3 showed a homogeneous phenotype (**A**). The osteogenesis potential was examined by alizarin red staining (**B**), and the adipogenesis was verified by oil red O staining (**C**). Alcian blue staining (**D**) was employed to assess the capacity for chondrogenesis. A colony formation assay using crystal violet staining revealed formation of a cell monoclonal colony after 7 days of culture (**E**,**F**). Specific cell surface antigen markers of SMSCs were tested thrice by flow cytometric analysis, and the representative results are shown (**G**). Magnification: A–C ×200, D ×40.

Primary SMSCs were derived from Wistar rat, and passage 3 of the SMSCs was used to characterise their self-renewal and differentiation ability as stem cells. Morphology of SMSCs at passage 3 became uniform, and the cells grew in a monolayer with typical fibroblast morphology (Fig. 1A). The results on the tri-lineage differentiation experiments showed that the SMSCs successfully differentiated into osteocytes (Fig. 1B), adipocytes (Fig. 1C), and chondrocytes (Fig. 1D). The self-renewal capacity of SMSCs was tested by colony formation assays (Fig. 1E,F). The colony formation index of SMSCs was 96.13% ± 2.06%. To verify the purity of SMSCs, we used flow cytometry to test cell surface antigen markers. The results revealed that the SMSCs consisted of a single phenotypic population positive for CD44 (98.64%), CD29 (98.23%), and CD90 (98.94%)^18–20^. By contrast, the cells were negative for other markers of the hematopoietic lineage, including lipopolysaccharide receptor CD34 (7.88%) and the leukocyte common antigen CD45 (4.03%), indicating their non-hematopoietic origin (Fig. 1G).

### Cell morphology and cell growth of MCs and SMSCs in the co-culture system

**Figure 2:**
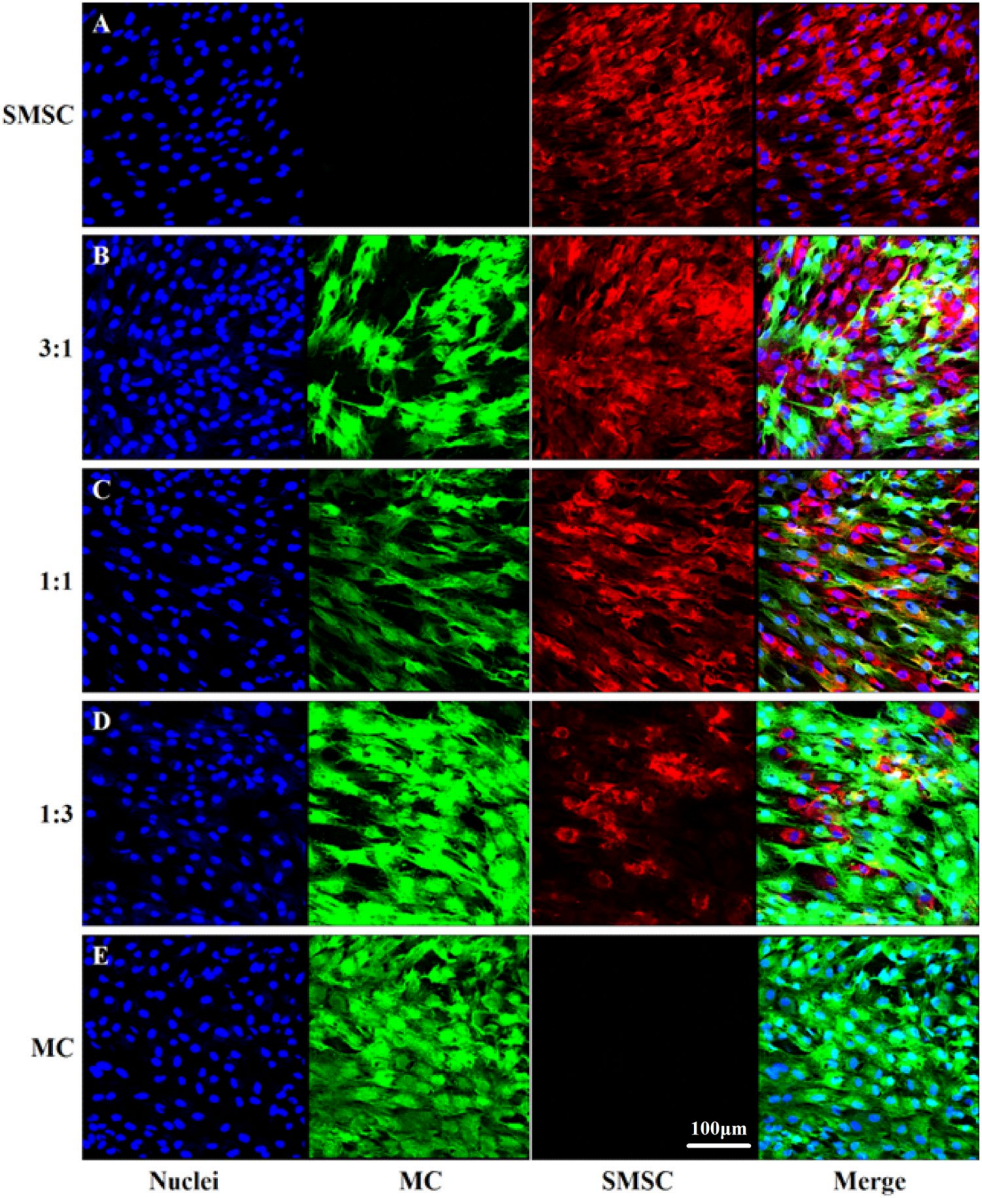
Cell morphology and cell growth in co-culture systems were examined by confocal laser scanning microscopy. MCs and SMSCs were stained with DiI and DiO, respectively, to display two colours (DiI: SMSCs, DiO: MCs). After co-culturing for 7 days, the two types of cells at different ratios reached confluence (**B**–**D**). In the co-culture groups, for example, a ratio of 3:1 means that the number of SMSCs was 3 times that of MCs. SMSCs had a spindle shape in the co-culture groups (red), while MCs had a polygonal shape both in the mono-culture control group and in the co-culture groups (green). Magnification: ×100.

Rat MCs cultured alone or co-cultured with fluorescently labelled SMSCs all reached confluence after 3 days culture. In all co-culture groups, two types of cells were well mixed with each other. SMSCs had a spindle shape in all co-culture groups (Fig. 2A–E),whereas MCs showed a round polygonal shape in the co-culture groups (Fig. 2B–D) and in the mono-culture control group (Fig. 2E). The cell numbers in the groups were: SMSC 1.32 ± 0.12e^5^/cm^2^, 3:1 1.37 ± 0.87e^5^/cm^2^, 1:1 1.29 ± 0.13e^5^/cm^2^, 1:3 1.28 ± 0.08e^5^/cm^2^, and MC 1.24 ± 0.06e^5^/cm^2^, respectively.

### The enhanced proliferation of chondrocytes in the co-culture system

**Figure 3:**
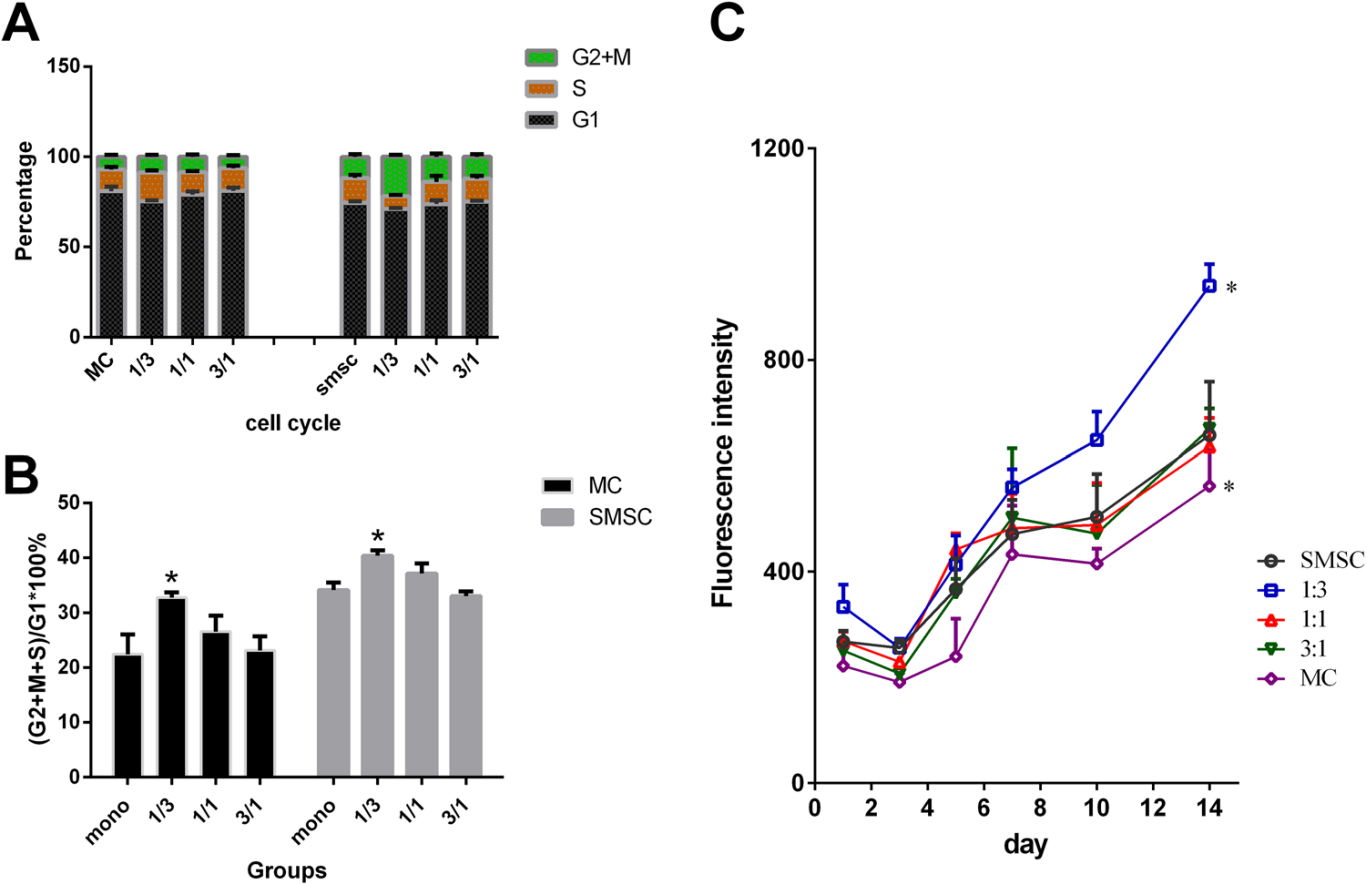
Cell cycle analysis was done after co-culturing for 1 day both in the mono-culture control group and in co-culture groups. Percentages of cells at each stage of the cell cycle were analysed (**A**). [(G2 + M/S)/G1] ×100% of cells in each group was calculated to evaluate the proliferation ratio (**B**). An Alamar Blue assay was used to determine the number of all cells in each group after co-culturing for different numbers of days (**C**).

To determine the proliferation ability of cells in the co-culture system, the percentage of SMSCs and MCs at each stage of the cell cycle was analysed respectively (Fig. 3A). The ratio (G2 + M/S)/G1was calculated to assess cell proliferation. Compared to the mono-cultured MC control group (22.43% ± 3.58%), the (G2 + M/S)/G1 rate of MCs increased in all co-culture groups (Fig. 3B). Compared to the mono-cultured SMSC group (34.14% ± 1.38%), the (G2 + M/S)/G1rate was 33.02% ± 0.88%, 37.13% ± 1.84% and 40.4% ± 0.95% when the SMSCs/MCs ratio was 3:1, 1:1 and 1:3, respectively. Results of the Alamar blue assay indicated that the number of cells increased over time for all groups, while the co-culture group with a SMSCs/MCs ratio of 1:3 yielded larger number of cells than others, with statistical significance on day 14 but no significant difference on day 1 or day 10 (Fig. 3C). Results of cell cycle analysis indicated that proliferation of MCs was promoted in the co-culture system on day 3, and after a long period of culture, cell numbers in all co-culture groups and the mono-cultured SMSC group were larger than the cell number in the mono-cultured MC group. However, no significant difference was detected between the co-culture group and SMSC mono-culture group, indicating that the co-culture system has a capacity for proliferation equivalent to that of SMSCs.

### Apoptosis analysis

**Figure 4:**
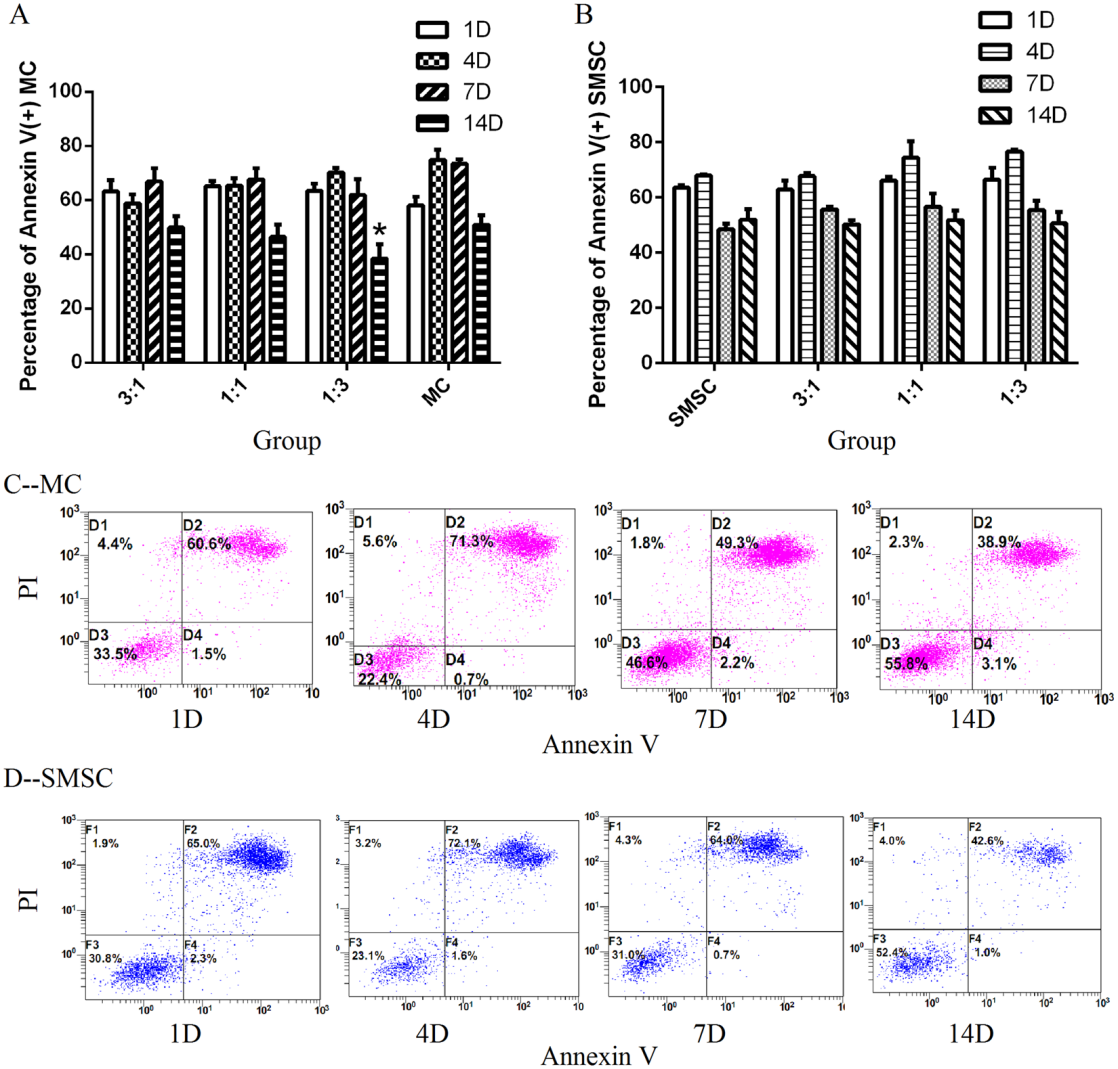
Cell apoptosis of SMSCs and MCs in different co-culture groups was analysed by Annexin V and PI staining. Intensity of fluorescence was detected by flow cytometry. MCs or SMSCs cultured for 1, 4, 7, or 14 days had different proportions of cells at the early (right lower quadrant in panels C, D) and late (right upper quadrant in panels C, D) stage of apoptosis. Data were analysed, and MCs and SMSCs are shown in panels (A) and (B) respectively (Three replicates, P < 0.05).

SMSCs were labelled with eFlour^®^ 450 and co-cultured with MCs for apoptosis detection. Apoptosis was analysed on days 1, 4, 7, and 14 by Annexin V and propidium iodide (PI) staining (Fig. 4C,D). Annexin V-positive cells corresponded to all cells at the early and late stage of apoptosis. MCs (Fig. 4A) and SMSCs (Fig. 4B) were analysed respectively in different groups. Results indicated that MCs in the 1:3 group had a lower ratio of apoptosis, and the difference was statistically significant. There was no significant difference for SMSCs between groups at different time points (days).

### Chondrogenic differentiation of the cells in the co-culture system

**Figure 5:**
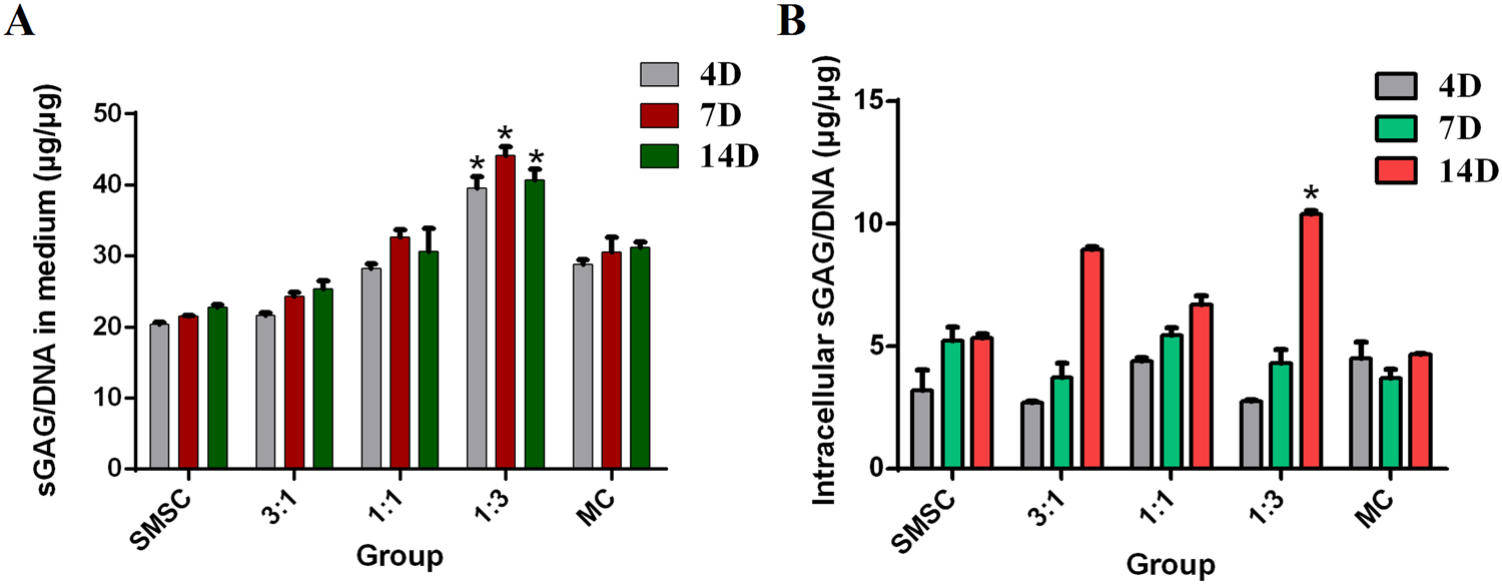
sGAG content of cells was quantified by means of the DMMB dye in the culture medium and inside cells, respectively (**A**,**B**). The ratio of GAG to DNA on different days was used for quantification of GAG (Three replicates, P < 0.05).

To evaluate the chondrogenic differentiation ability, sulphated glycosaminoglycan (sGAG) levels in the culture medium and inner cells of all groups were evaluated by means of the DMMB reagent at different time points. In the culture medium, the co-culture group at 1:3 ratio showed the highest sGAG secretion level at all time points (Fig. 5A). In the cells, the sGAG level showed no significant differences among groups except for the group with 1:3 ratio on day 14 (Fig. 5B). These results indicated better chondrogenic differentiation ability of cells in co-culture group at 1:3.

### The gene expression profile of MCs in the co-culture system

**Figure 6:**
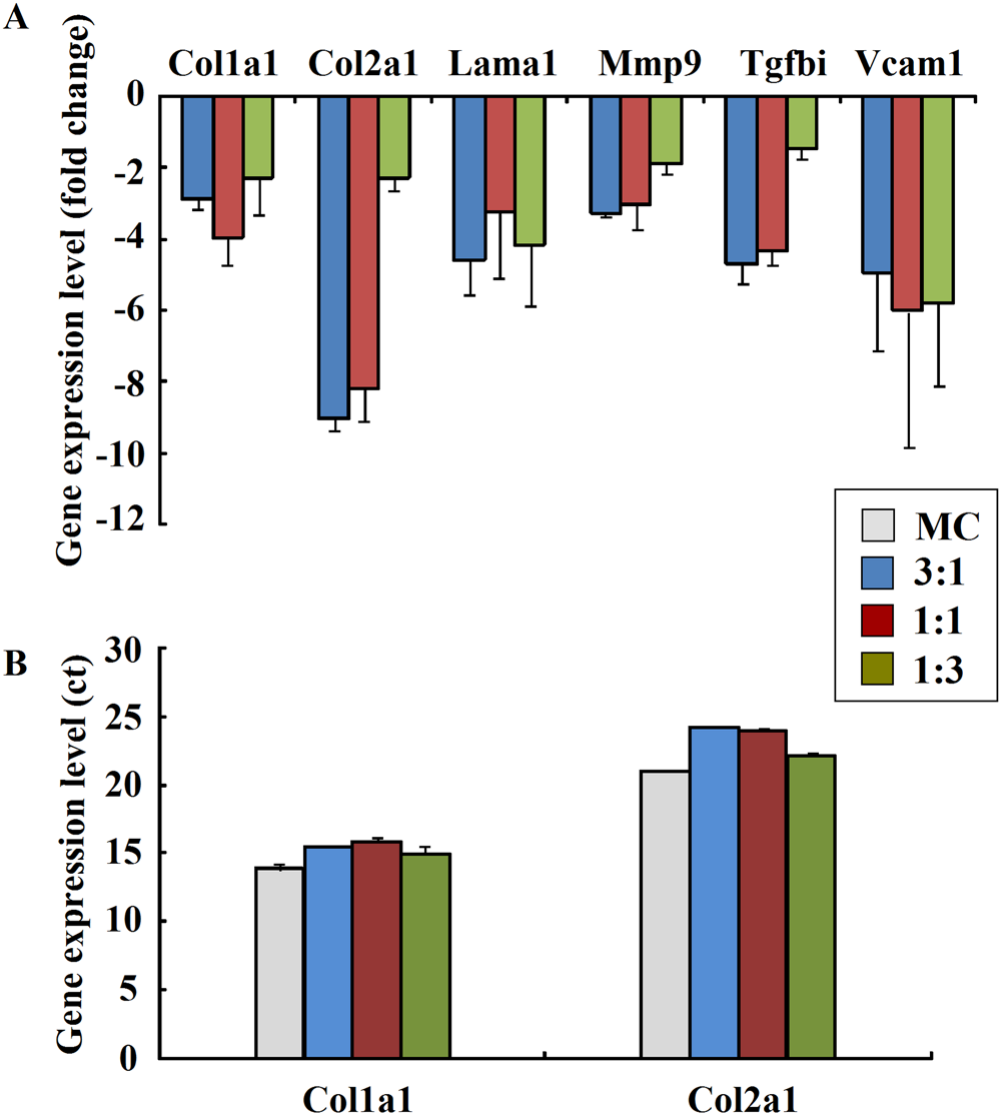
The ECM gene chip analysis showed different expression in MCs between different co-culture groups and passage 1 of MC cells after 7 days. Genes that were significantly differentially expressed among the groups are displayed. Mono-cultured MCs at passage 1 served as the control group (**A**). Ct values of *col1a1* and *col2a1* in each group were shown (**B**). (Three replicates, P < 0.05).

The ECM chip was analysed with the 96-well rat ECM and the PCR array of adhesion molecules. Among all the 96 genes of the PCR array (Table 1), six genes were found to be expressed significantly different between co-culture groups and the mono-cultured MC group, namely *col1a1*, *col2a1*, *lama1*, *mmp9*, *tgfbi*, and *vcam1*. The folds of gene expression level were shown while MC control was set as baseline (Fig. 6A). Among them, *col2a1*, *col1a1*, and *lama1* encode collagens and ECM constituents, *mmp9* encodes an ECM protease, and *tgfbi* codes for an adhesion-related molecule. Ct values of *col1a1* and c*ol2a1*genes were shown (Fig. 6B). Cells in 1:3 group expressed more type 1 and 2 collagen comparing to other two co-culture groups, and were more closer to MC. These results indicated that co-culture at 1:3 ratio can help maintain collagen expression of MC *in vitro*.

**Table 1:** RT² Profiler™ PCR Array of Rat Extracellular Matrix & Adhesion Molecules.

Adamts1	Adamts2	Adamts5	Adamts8	Catna1	Cd44	Cdh1	Cdh2	Cdh3	Cdh4	Cntn1	**Col1a1**
Col2a1	Col3a1	Col4a1	Col4a2	Col4a3	Col5a1	Col6a1	Col8a1	Ctgf	Ctnna2	Ctnnb1	Ecm1
Emilin1	Entpd1	Fbln1	Fn1	Hapln1	Icam1	Itga2	Itga3	Itga4	Itga5	Itgad	Itgae
Itgal	Itgam	Itgav	Itgb1	Itgb2	Itgb3	Itgb4	**Lama1**	Lama2	Lama3	Lamb2	Lamb3
Lamc1	Mmp10	Mmp11	Mmp12	Mmp13	Mmp14	Mmp15	Mmp16	Mmp1	Mmp2	Mmp3	Mmp7
Mmp8	**Mmp9**	Ncam1	Ncam2	Pecam1	Postn	Sele	Sell	Selp	Sgce	Sparc	Spock1
Spp1	Syt1	**Tgfbi**	Thbs1	Thbs2	Timp1	Timp2	Timp3	Tnc	**Vcam1**	Vcan	Vtn
Actb	B2m	Hprt1	Ldha	Rplp1	RGDC	RTC	RTC	RTC	PPC	PPC	PPC

Genes were shown in bold while its expression level had significant difference. P < 0.05.

## Discussion

Tissue engineering of meniscus requires large amounts of cells and secretion of ECM. Poor proliferation capacity of the meniscal cells highly limits their applications to meniscus regeneration. In contrast, MSCs have excellent proliferation ability both *in vitro* and *in vivo*. MSCs have been used as a popular source of seed stem cell to reconstruct different kinds of tissues in recent years. How to induce MSCs to differentiate into MCs which are heterogeneous in nature remains unknown. We hypothesised that the co-culture system of SMSCs and autologous MCs might be a solution to the problem.

In this study, SMSCs were chosen because they are believed to have better proliferation and chondrogenic differentiation abilities than BMMSCs do^21,22^. Ding Z *et al*. proved that MSCs in the meniscus have a pronounced tendency for chondrogenic differentiation, while BMMSCs showed a significantly greater osteogenic potential, *in vitro* and *in vivo*^23^. Meanwhile SMSCs were reported to perform an important function in meniscus regeneration and differentiation *in vivo*^11,24,25^. According to the literature and our previous studies, SMSCs showed good capacity for self-renewal and proliferation. They can be expanded to generation 5 or 8 without apparent dedifferentiation. Passage 3 SMSCs, as used in our experiment, have a homogeneous appearance and a high degree of purity. If we applied different culture media, SMSCs could differentiate into osteoblasts, lipoblasts, or chondrocytes. All these results confirmed SMSCs as a suitable source of seed cells.

Our results revealed that more SMSCs transitioned to the mitotic stage when co-cultured with MCs after 1 day of co-culture. With the ratio of SMSCs/MC at 1:3, SMSCs showed the highest proliferation ability, while the largest number of MCs staying in the S phase was detected. Thus, our results indicated that at the ratio of SMSCs/MCs of 1:3, both SMSCs and MCs had the highest proliferation ability. During the assessment of the two types of cells as a whole, a larger number of cells were observed in the co-cultured groups than in the mono-cultured MC group. Cell numbers of co-culture groups were larger than that in the MC mono-culture group and were always roughly equal to that in the SMSC mono-culture group. The proliferation ability of both two types of cells was promoted in the co-culture system. There is no conclusive evidence whether the proliferation boost received by the MSCs is mediated by direct cell**–**cell contacts or secreted soluble mediators^26,27^. Further studies are warranted to determine the underlying mechanism.

Differentiation capacity is another important parameter for evaluation of the seed cells for tissue engineering. In this study, we analysed and quantified the sGAG content of the co-culture system. Although meniscal cells expressed different types of matrix genes as compared to chondrocytes, we used the chondrogenic differentiation medium because there was no medium available that can induce MSCs to differentiate into the meniscus only. Our results showed that all the co-cultured groups (especially the group with the ratio of SMSCs/MCs at 1:3) showed more sGAG secretion than the mono-culture group did. Our results are consistent with findings in other studies in that the ECM gene expression of MCs can be influenced by co-culture with MSCs^28–31^. Another study, which was based on BMMSCs instead of SMSCs, showed similar results and indicated that a co-culture ratio BMMSCs/MCs at 1:3 yields the highest production of type I collagen and sGAG as well as the lowest expression of hypertrophic genes, such as *col10a1* and *mmp13*^16^. While in a 3D culture study, a ratio of 75:25 or 100:0 (MSC mono-culture) culture groups produced significantly more GAG/DNA than meniscal fibrochondrocytes in mono-culture. GAG retention was highest in 50:50 co-cultures^32^. That might indicate that there are differences between plate culture and 3D culture, which requires further study for verification.

The gene chip results indicated that the collagens and ECM constituents such as *col2a1*, *col1a1*, and *lama1*, ECM protease *Mmp9*, as well as the adhesion-related molecule *Tgfbi* were significant differentially expressed between co-culture groups and mono-culture groups. Passage 3 of MCs was used in the co-culture groups. To evaluate their ECM and adhesion molecule expression levels compared with normal MCs, we used passage 1 MCs as a control group. The smallest difference was observed between the 1:3 SMSCs/MC co-culture group and the passage 1 MC group. MCs are mixture of two types, fibroblast-like and chondrocyte-like, represented by collagen I and II expression. In our results, *col2a1*and *col1a1* genes were down-regulated in passage 3 MC compared to passage 1, which indicated long time culture will weaken the expression of both two genes. However, the 1:3 co-culture group could maintain the cell phenotype. *Tgfbi* encodes an RGD-containing protein that binds to type I, II and IV collagens. The protein is induced by transforming growth factor-beta and acts to inhibit cell adhesion. While the enzyme encoded by *Mmp9* degrades type IV and V collagens. Fuller ES *et al*. revealed that metalloproteinase expression was different in different zones of meniscus: ADAMTS4, MMP1, MMP3 were upregulated more by IL-1α in inner zones whereas ADAMTS5, MMP13 and MMP9 were more upregulated by TNFα in outer zones^33^. It has shown the abilities of cell adhesion and matrix degradation were different between co-culture groups.

In summary, the co-culture system of SMSCs/MCs at the ratio of 1:3 promotes proliferation of both MCs and SMSCs and increases the total secreted amount of chondrogenic differentiation marker sGAG with minimal altering expression of ECM genes in MCs. These results suggest that co-culture of SMSCs play a positive role in the maintenance of the MC phenotype and matrix gene expression; these benefits make the co-culture of MCs and SMSCs a promising strategy for meniscus repair.

## Materials and Methods

### Cell culture

SMSCs were derived from knees of Wistar rats with the approval of the Animal Ethics Committee of Peking University Third Hospital (Beijing, China), and all the animal experiments were carried out in compliance with the Guide for the Care and Use of Laboratory Animals (National Academy Press, NIH Publication No. 85-23, revised 1996). Rats were euthanised using an intraperitoneal injection of 20% urethane, after which their lower extremities were isolated. After the skin and muscles were dissected, the suprapatellar bursa was exposed by cutting the patella tendon. The synovium on the femoral condyle was harvested carefully, and then flushed using phosphate-buffered saline (PBS) supplemented with 100 μg/ml penicillin and streptomycin into a 100-mm culture dish. Meniscus were harvested and washed by PBS for 3 times. The synovium was digested with collagenase I for 2 h at 37 °C to dissociate cells, which were centrifuged, counted, and seeded at a density of 10^6^ in 8 ml of α-minimal essential medium with 10% of foetal bovine serum (Gibco, Carlsbad, CA, USA), 100 U/ml penicillin, and streptomycin (Gibco) per 100-mm culture dish. While MCs were digested by collagenase I and II for 2 h before centrifuge, followed by the same subsequent processing as SMSCs. The cells were then cultured at 37 °C in a humidified atmosphere of 95% air and 5% CO_2_ and any non-adherent cells were removed 48 h after seeding by changing the medium. Thereafter, the medium was replaced every 3 days. After 7 days of primary culture, cells grown to 90% confluence were digested with 0.25% trypsin/1 mM EDTA, and cells between the primary and third passages were used for further experiments. Different ratio of SMSCs and MC were counted by cytometer and mixed before seeding. Cells of 10^4^ per cm^2^ were seeded in cell culture dish. In all groups, total cell number was identical.

### Identification of the characteristics of SMSCs

The specific cell surface antigen markers of SMSCs were examined by flow cytometry. The antibodies for positive markers included CD44 (ab51037), CD90 (ab23894), and CD29 (ab52971)^20,34,35^, whereas the negative markers included CD34 (ab6330) and CD45 (ab33533) (Abcam). Antibody for IgG was used as a negative control. FITC-labelled antibodies specifically bound to the SMSC surface, and positive cells were detected by flow cytometry. Three replicates for each marker and the positive ratio were analysed by means of BD FACSuite Software Application. A trilineage-induced differentiation experiment was also conducted to identify the multiple differentiation potential of SMSCs, including adipogenesis, osteogenesis, and chondrogenesis. SMSCs of passage 3 were used in this experiment. Briefly, the SMSCs were incubated in a 6-well plate at a density of 10^5^ cells per well with the rat MSC Adipogenic or Osteogenic Differentiation Medium (Cyagen Biosciences Inc, Sunnyvale, CA, USA) for adipogenesis or osteogenesis induction, respectively. The cells were then examined for adipogenesis by oil red O staining after a week of culture or for osteogenesis by alizarin red staining after 2 weeks of cultivation. For analysis of chondrogenesis, a micromass technique was adapted from Ahrens *et al*.^36,37^. Briefly, SMSCs of passage 3 were trypsinised and resuspended in the growth medium at a density of 100,000 cells per 10 μl. Ten-microliter droplets were seeded in culture dishes and allowed to form cell aggregates and substratum at 37 °C for 2.5 h. The chondrogenic medium (consisting of Dulbecco’s modified Eagle’s medium [DMEM], 1% of foetal bovine serum (FBS), 1% of penicillin/streptomycin, 37.5 μg/ml ascorbate-2-phosphates, 1% of ITS premix, and 10 ng/ml TGF-β1) was carefully added around the cell aggregates. The chondrogenic medium was replenished every 3 days. Micromasses were stained with Alcian blue to evaluate the chondrogenic differentiation at 7, 14, 21 days of culture. Alcian blue staining for GAG was performed to determine whether the cells functioned as chondrocytes.

A colony formation assay using crystal violet staining showed cell monoclonal colony formation after 7 days of culture. A total of 100, 200, and 300 cells were seeded in a 100-mm culture dish. The culture medium was changed every 3 days. After 7 days, the resulting colonies were fixed with methanol at −20 °C for 5 min, and then stained with crystal violet. Colonies with a diameter greater than 4.5 mm or with more than 10 cells were counted and were analysed in the Image-Pro Plus (IPP 6.0) software. The colony formation index was defined as the ratio of colony numbers to initial numbers of the cells plated. All the experiments were done in triplicate, and three independent repeated experiments were performed.

### Cell cycle analysis and proliferation assay

Passage 3 SMSCs were labelled with 5,6-caboxyfluorescein diacetate, succinimidyl ester (CFDA SE; Invitrogen) immediately prior to co-culture. The labelling procedure was performed according to the manufacture’s protocol at a final dye concentration of 10 μM. Direct co-cultures of SMSCs and MCs were performed in a monolayer at different ratios in DMEM (Gibco) supplemented with 10% of FBS (Gibco), 100 U/ml penicillin and 100 μg/ml streptomycin. CFDA SE-labelled SMSCs and unlabelled MCs were cultured alone and served as control groups, respectively. After 1 day of culture, the cells were trypsinised into a single-cell suspension for cell cycle analysis. The cells were trypsinised and passed through a 40-μm filter (BD Biosciences) to remove cell clumps. After washed by cold PBS for three times, 10^6^ cells was resuspended in 1 ml PBS. Ethyl alcohol was blending into cell suspension on ice to form final volume of 3.5 ml. Then cells were cultured with RNAase for 30 min, and one drop of PI (0.5 mg/ml, 1% Triton-100) was added prior to flow cytometer analysis. The cell cycle data were calculated and matched by means of ModFit LT 4.0. An Alamar Blue assay was used to evaluate cell proliferation and viability. On days of 1, 3, 5, 7, 10 and 14, the culture medium was removed and replaced with 1 ml of a serum-free medium containing 10% of the Alamar Blue stock solution (Invitrogen, Carlsbad, CA, USA). The plates were then incubated at 37 °C for 3 h. An aliquot of 100 μl from each well was transferred to a 96-well plate, and the fluorescence intensity was read at *λ*_ex_ = 570 nm and *λ*_em_ = 585 nm on a fluorescence microplate reader (FlexStation 3, Molecular Devices, Union City, CA, USA). After removing the remaining Alamar Blue working solution from the micromass-containing 12-well plates, fresh culture medium was added and the systems were further incubated.

### Confocal microscopy examination of morphology of co-cultured cells

To examine the cell morphology, SMSCs and MCs were stained with DiI and DiO dye (Molecular Probes), respectively. The labelling procedure was performed according to the manufacturer’s instructions. The cells were digested and incubated with a DiI or DiO solution for 30 min at 37 °C and then mixed together for further cultivation. After co-culture for 7 days, images of SMSCs and MCs at different ratios were captured by means of a TCS SP5 confocal laser scanning microscope (Leica) using 4 fluorescent channels according to the excitation/emission spectra of the dyes. Five pictures in each group were taken randomly at magnification 200x.

### Apoptosis detection by Annexin V and PI

SMSCs were labelled with eFlour^®^ 450 as manufacturer’s instruction. After cultivation for various periods, the cells were digested and pelleted by centrifugation, washed twice with ice-cold PBS, and resuspended in binding buffer (10 mM HEPES-NaOH [pH 7.4], 140 mM NaCl, and 2.5 mM CaCl_2_) to a concentration of 10^6^/ml. Next, 0.1 ml of this cell suspension was transferred to a 5-ml tube and incubated with 0.005 ml of annexin V and 0.005 ml of PI for 15 min at 25 °C in the dark. Finally, 0.4 ml of binding buffer was added, and the samples were analysed by flow cytometry within 1 h on a Gallios flow cytometer (Beckman Coulter). The samples were gated on the basis of forward versus side scatter for size, and the results are presented as the percentage of cells that were viable (Ann-V^−^PI^−^), early apoptotic (Ann-V^+^PI^−^), or nonviable (Ann-V^+^PI^+^ or Ann-V^−^PI^+^).

### The chondrogenic differentiation ability of the co-culture system

The micromass culture technique was used in this study to improve chondrogenic efficiency. The micromass technique was modified from the method of Ahrens *et al*. Briefly, cells from the third passage were harvested and resuspended in a growth medium at 10^7^ cells/ml. Ten-microliter droplets were placed in culture dishes, and the cells were allowed to adhere to each other and the substratum at 37 °C for 90 min. After that, the chondrogenic medium consisting of DMEM, 1% of FBS, 1% of penicillin/streptomycin, 37.5 mg/ml ascorbate-2-phosphate, insulin/transferrin/selenium premix (BD Biosciences, Bedford, MA), and 10 ng/ml transforming growth factor-β1 (Research Diagnostics Inc., Flanders, NJ) were carefully added around the cell aggregates. At 24 h, the cell aggregates coalesced and became spherical. The chondrogenic medium was changed every 3 days. The culture media were collected and stored at −80 °C and the micromasses were harvested for DNA at various time points.

The DNA and sGAG were quantified using a Varioskan Flash reader. Both the amount of sGAG in cells and that released into the culture medium were examined. The amount of DNA was measured by the Hoechst 33258 fluorometric assay (Polysciences Inc., Warrington, PA, USA). Briefly, 20 μl of the cell solution was incubated at 37 °C for 1 h with 200 μl of a Hoechst 33258 working solution (2 mg/μl). The fluorescence intensities were determined at 360 nm (excitation) and 460 nm (emission). The DNA content was determined using a standard curve of calf thymus DNA (Sigma, St. Louis, MO, USA; R^2^ = 0.9992). Proteoglycan content was estimated from the sGAG content using a 1,9-dimethylmethylene blue (DMMB; Sigma, St. Louis, MO, USA) dye-binding assay. Cells were digested with papain and 20 μl of a cell lysis solution was mixed with 200 μl of the DMMB reagent, and the absorbance values were measured on a plate reader at 525 nm. To detect extracellular sGAG content (released), 20 μl culture medium was collected. A standard curve based on chondroitin-6-sulphate from shark (Sigma, St. Louis, MO, USA) was constructed to determine the sGAG content (R^2^ = 0.9956).

### Flow cytometry for cell sorting

Cells were co-cultured for 7 days before fluorescence activated cell sorting (FACS; BD FACS AriaTM SORP) was carried out for gene expression analysis. Briefly, SMSCs were labelled with CDFA SE (Invitrogen) before co-culture. Then, all the cells were trypsinised and passed through a 40-μm filter (BD Biosciences) to remove cell clumps prior to flow cytometer sorting. The P1 Gate was set to exclude all dead cells, cell debris, and cell clumps. Next, gated cells were excited at 488 nm with a green emission filter to be further gated into the P2 Gate as CDFA-SE-positively-labelled SMSCs and P3 Gate as CFDA-SE-unlabelled MCs.

### PCR Chip of the ECM and adhesion factors

After co-cultivation for 7 days, the total RNA of MCs was extracted by means of the TRIzol Reagent (Invitrogen) and reverse-transcribed using the M-MLV kit (Qiagen, Hilden, Germany) according to the manufacturer’s instructions. We selected RT² Profiler™ PCR Array Rat Extracellular Matrix & Adhesion Molecules (PARN-013Z, Qiagen) to screen for differentially expressed genes between the co-culture group and MC group. All the genes provided by the manufacturer are shown in Table 1. Real-time PCR was run by a technician. Data were analysed as follows: ΔΔCt was calculated for each gene across two PCR Arrays (or groups) as ΔΔCt = ΔCt_group2_ − ΔCt_group1_ where group 1 is the control group and group 2 is the experimental group. The fold change for each gene from group 1 to group 2 was determined as 2−ΔΔCt. Three replicates of each group were used in this experiment. Ct values of *col1a1* and *col2a1* were shown in different samples. In the results, bigger Ct value indicated lower expression level of each gene.

### Statistical analysis

All experiments were repeated at least three times. Data were presented as the mean ± SD of three experiments. Statistical significance was determined by one-way or two-way ANOVA. Pair wise differences between groups were analysed, and *P* values less than 0.05 were assumed to indicate significance. In PCR array results, if the fold change was greater than 1.0, then the result was reported as a fold up-regulation. If the fold change was less than 1.0, then the negative inverse of the result was reported as a fold down-regulation.

## Electronic supplementary material

Dataset 1
