# Supplementary material for: A co-culture system of rat synovial stem cells and meniscus cells promotes cell proliferation and differentiation as compared to mono-culture
Source: Sci Rep. 2018 May 16;8:7693. doi: 10.1038/s41598-018-25709-w (PMC5955983; doi:10.1038/s41598-018-25709-w)
Supplement: Supplementary file 1 — Dataset 1 [file 41598_2018_25709_MOESM1_ESM.docx]

**A co-culture system of rat synovial stem cells and meniscus cells promotes cell proliferation and differentiation as compared to mono-culture**

Xing Xie^*, 1^, Jingxian Zhu^*, 1^, Xiaoqing Hu^1^, Linghui Dai^1^, Xin Fu^1^, Jiying Zhang^1^, Xiaoning Duan^1^, Yingfang Ao**^,1^

1. Institute of Sports Medicine, Beijing Key Laboratory of Sports Injury, Third Hospital of Peking University

*These authors contributed equally to this work. **Corresponding Author

Correspondence to aoyingfang@163.com

| Group | Gene | Adamts1 | Adamts2 | Adamts5 | Adamts8 | Catna1 | Cd44 | Cdh1 | Cdh2 | Cdh3 | Cdh4 | Cntn1 | Col1a1 |
| --- | --- | --- | --- | --- | --- | --- | --- | --- | --- | --- | --- | --- | --- |
| 3:1 | Average ct | 21.35427 | 22.14111 | 21.17676 | 30.41898 | 20.54245 | 24.43169 | 37.82348 | 27.20798 | 26.40557 | 33.60589 | 35.76539 | 15.39806 |
|  | std | 0.044404 | 0.254853 | 0.294822 | 0.333941 | 0.080179 | 0.104879 | 0.020909 | 0.202502 | 0.116698 | 0 | 0.445779 | 0.142459 |
| 1:1 | Average ct | 21.94361 | 22.87237 | 21.78732 | 31.41721 | 21.05725 | 24.90608 | 39.35728 | 27.67455 | 26.70942 | 31.73482 | 35.50494 | 15.83364 |
|  | std | 0.070116 | 0.205001 | 0.493604 | 0.427873 | 0.163749 | 0.605406 | 0 | 0.346143 | 0.012833 | 0 | 0.238945 | 0.269907 |
| 1:3 | Average ct | 20.90491 | 22.33801 | 21.1865 | 30.33969 | 20.09357 | 23.22293 | 32.96835 | 27.32783 | 25.45274 | 33.05574 | 33.81776 | 14.99275 |
|  | std | 0.091328 | 0.190777 | 0.162433 | 0.921389 | 0.315233 | 0.248529 | 0.300414 | 0.343288 | 0.461055 | 0 | 1.63254 | 0.575673 |
| MC | Average ct | 19.92838 | 21.90149 | 20.28693 | 30.14195 | 19.45223 | 23.16682 | 33.53155 | 27.12931 | 24.75097 | 33.46413 | 32.39049 | 13.87328 |
|  | std | 0.391452 | 0.204013 | 0.306831 | 0.521998 | 0.297112 | 0.243122 | 0.846907 | 0.488515 | 0.229399 | 0.654796 | 0.749705 | 0.38565 |
|  |  | Col2a1 | Col3a1 | Col4a1 | Col4a2 | Col4a3 | Col5a1 | Col6a1 | Col8a1 | Ctgf | Ctnna2 | Ctnnb1 | Ecm1 |
| 3:1 | Average ct | 24.15006 | 16.62623 | 20.03037 | 21.21573 | 30.51593 | 20.79301 | 19.90295 | 25.42592 | 18.76452 | 0 | 20.77069 | 24.83894 |
|  | std | 0.060683 | 0.119147 | 0.155762 | 0.064478 | 0.361343 | 0.177247 | 0.125965 | 0.100814 | 0.154593 | 0 | 0.105371 | 0.2668 |
| 1:1 | Average ct | 24.00636 | 17.20458 | 21.16834 | 22.19739 | 32.46127 | 21.63177 | 20.48162 | 26.30118 | 19.11837 | 33.52566 | 20.76492 | 24.42733 |
|  | std | 0.164933 | 0.320955 | 0.248101 | 0.098621 | 0.764159 | 0.331973 | 0.344106 | 0.165989 | 0.2437 | 1.096964 | 0.112097 | 0.77129 |
| 1:3 | Average ct | 22.18266 | 16.78135 | 21.08185 | 21.66337 | 30.3626 | 20.82417 | 19.11919 | 25.05942 | 17.7577 | 32.79247 | 19.52622 | 23.84429 |
|  | std | 0.212962 | 0.308676 | 0.143486 | 0.193547 | 0.085271 | 0.287556 | 0.373033 | 0.164379 | 0.092881 | 0.449738 | 0.239512 | 0.605039 |
| MC | Average ct | 20.98278 | 16.18667 | 20.70283 | 21.28321 | 30.11276 | 20.04817 | 18.56788 | 24.99178 | 17.28378 | 32.07548 | 18.78155 | 23.43723 |
|  | std | 0.081832 | 0.362095 | 0.330856 | 0.074877 | 0.389256 | 0.292667 | 0.192782 | 0.497561 | 0.20237 | 0.183547 | 0.385746 | 0.21991 |
|  |  | Emilin1 | Entpd1 | Fbln1 | Fn1 | Hapln1 | Icam1 | Itga2 | Itga3 | Itga4 | Itga5 | Itgad | Itgae |
| 3:1 | Average ct | 26.39166 | 34.68191 | 25.1071 | 17.2277 | 26.51952 | 22.14401 | 26.72129 | 24.17505 | 29.04648 | 22.76176 | 32.06173 | 29.51237 |
|  | std | 0.243742 | 0.429337 | 0.174158 | 0.060563 | 0.254964 | 0.120644 | 0.091019 | 0.182615 | 0.188847 | 0.107284 | 0.389265 | 0.205139 |
| 1:1 | Average ct | 26.51622 | 33.13066 | 25.91118 | 17.60339 | 26.49628 | 22.35786 | 27.15865 | 25.00855 | 29.08616 | 23.24685 | 32.44631 | 29.5759 |
|  | std | 0.347504 | 0.292536 | 0.111002 | 0.235993 | 0.300455 | 0.104567 | 0.185372 | 0.403336 | 0.253963 | 0.37578 | 0.185886 | 0.508198 |
| 1:3 | Average ct | 25.25497 | 32.59523 | 25.68392 | 15.94967 | 24.74166 | 20.86138 | 26.1891 | 23.53083 | 28.35311 | 22.37165 | 32.12544 | 31.18023 |
|  | std | 0.252511 | 0.824269 | 0.166809 | 0.086025 | 0.121634 | 0.162057 | 0.19643 | 0.192131 | 0.297549 | 0.14777 | 0.506343 | 0.521286 |
| MC | Average ct | 24.19704 | 31.77444 | 25.51566 | 15.24862 | 23.57715 | 20.5112 | 25.61981 | 23.23807 | 27.38896 | 21.71986 | 31.24657 | 30.63859 |
|  | std | 0.19476 | 0.313144 | 0.311535 | 0.455855 | 0.255686 | 0.195239 | 0.260133 | 0.305494 | 0.327319 | 0.33929 | 0.670798 | 0.073829 |
|  |  | Itgal | Itgam | Itgav | Itgb1 | Itgb2 | Itgb3 | Itgb4 | Lama1 | Lama2 | Lama3 | Lamb2 | Lamb3 |
| 3:1 | Average ct | 31.77608 | 32.04354 | 22.32671 | 20.78561 | 26.62011 | 28.66792 | 33.88418 | 34.86706 | 26.97672 | 30.89571 | 23.51512 | 28.88853 |
|  | std | 0.670523 | 0.366699 | 0.105149 | 0.166117 | 0.345597 | 0.12628 | 0.564039 | 0.632149 | 0.119121 | 0.070572 | 0.278886 | 0.327145 |
| 1:1 | Average ct | 31.3545 | 32.52915 | 22.86604 | 21.83112 | 26.67569 | 28.99814 | 34.03652 | 33.69525 | 27.42027 | 30.31272 | 24.58447 | 28.84352 |
|  | std | 0.933191 | 0.58549 | 0.294652 | 0.445049 | 0.28929 | 0.410848 | 0.233024 | 0.832815 | 0.09536 | 0.137067 | 0.303366 | 0.194104 |
| 1:3 | Average ct | 29.76594 | 32.62395 | 21.92992 | 20.82243 | 26.31363 | 28.35736 | 34.83241 | 34.38864 | 27.32821 | 29.80085 | 24.22267 | 28.30435 |
|  | std | 0.560417 | 0.424946 | 0.023018 | 0.260396 | 0.134181 | 0.180306 | 0.939997 | 0.878927 | 0.236596 | 0.306961 | 0.232086 | 0.410191 |
| MC | Average ct | 28.59725 | 31.45965 | 21.12767 | 19.97006 | 25.25962 | 27.88117 | 34.64436 | 32.63505 | 26.73204 | 29.16325 | 23.439 | 27.74929 |
|  | std | 0.695391 | 0.529992 | 0.398171 | 0.359255 | 0.395607 | 0.193701 | 0.633591 | 0.241326 | 0.360974 | 0.480814 | 0.465229 | 0.308343 |
|  |  | Lamc1 | Mmp10 | Mmp11 | Mmp12 | Mmp13 | Mmp14 | Mmp15 | Mmp16 | Mmp1 | Mmp2 | Mmp3 | Mmp7 |
| 3:1 | Average ct | 20.92274 | 32.30137 | 23.36272 | 23.2619 | 18.7845 | 19.4534 | 29.72743 | 24.84468 | 38.86708 | 18.23598 | 19.51959 | 31.38068 |
|  | std | 0.257567 | 0.149413 | 0.070386 | 0.155215 | 0.218934 | 0.074256 | 0.037951 | 0.09027 | 0 | 0.181285 | 0.210488 | 1.075411 |
| 1:1 | Average ct | 21.43805 | 32.12152 | 23.82926 | 23.04125 | 18.7111 | 19.9282 | 30.36718 | 25.21519 | 0 | 19.01464 | 19.64887 | 31.92899 |
|  | std | 0.500669 | 0.118165 | 0.048591 | 0.338226 | 0.170095 | 0.344809 | 0.625679 | 0.1608 | 0 | 0.33287 | 0.046765 | 0.844008 |
| 1:3 | Average ct | 20.67439 | 30.90052 | 23.40958 | 22.56168 | 17.83194 | 18.74285 | 28.91916 | 23.74687 | 38.27159 | 18.17465 | 18.71709 | 31.21195 |
|  | std | 0.28256 | 0.550618 | 0.227509 | 0.173183 | 0.181572 | 0.194348 | 0.222325 | 0.178187 | 0.748456 | 0.23281 | 0.169387 | 0.931876 |
| MC | Average ct | 20.3746 | 29.83327 | 23.28605 | 21.89702 | 17.29542 | 18.12724 | 28.3418 | 23.04829 | 36.78502 | 17.45197 | 18.01606 | 30.22746 |
|  | std | 0.39561 | 0.442943 | 0.195403 | 0.318216 | 0.363556 | 0.309191 | 0.554347 | 0.227089 | 0.163888 | 0.511561 | 0.333448 | 0.182444 |
|  |  | Mmp8 | Mmp9 | Ncam1 | Ncam2 | Pecam1 | Postn | Sele | Sell | Selp | Sgce | Sparc | Spock1 |
| 3:1 | Average ct | 0 | 28.47464 | 21.68799 | 33.07421 | 36.31948 | 20.93321 | 0 | 34.20748 | 33.26797 | 22.81761 | 17.4832 | 27.58542 |
|  | std | 0 | 0.025032 | 0.038865 | 0.05401 | 2.755706 | 0.20938 | 0 | 0.446576 | 0.057108 | 0.389091 | 0.046965 | 0.364051 |
| 1:1 | Average ct | 0 | 28.32928 | 22.15755 | 32.52237 | 34.95063 | 21.6313 | 0 | 34.70346 | 33.25518 | 23.30532 | 17.59694 | 27.96896 |
|  | std | 0 | 0.325932 | 0.147475 | 0.577908 | 1.260484 | 0.265881 | 0 | 0.159198 | 0.167477 | 0.191433 | 0.400671 | 0.526062 |
| 1:3 | Average ct | 32.89171 | 27.6388 | 20.86407 | 34.7306 | 33.91327 | 21.52359 | 34.11143 | 35.06398 | 34.02715 | 22.85333 | 16.26032 | 27.17095 |
|  | std | 1.017303 | 0.208956 | 0.075018 | 0.285731 | 0.271478 | 0.153997 | 0 | 0 | 0.340538 | 0.252408 | 0.152445 | 0.094867 |
| MC | Average ct | 31.86239 | 26.75297 | 20.23075 | 30.86567 | 32.68363 | 20.43747 | 32.72083 | 34.51404 | 32.03404 | 21.92254 | 16.03322 | 26.31596 |
|  | std | 0.232519 | 0.337289 | 0.275015 | 0.618963 | 0.479158 | 0.333252 | 0.378673 | 1.057664 | 0.628347 | 0.536444 | 0.16722 | 0.608123 |
|  |  | Spp1 | Syt1 | Tgfbi | Thbs1 | Thbs2 | Timp1 | Timp2 | Timp3 | Tnc | Vcam1 | Vcan | Vtn |
| 3:1 | Average ct | 21.1809 | 33.77222 | 22.71924 | 17.23946 | 18.55954 | 18.54267 | 18.45261 | 23.40228 | 21.31753 | 27.75173 | 21.05212 | 33.42186 |
|  | std | 0.607472 | 0 | 0.16778 | 0.084604 | 0.147399 | 0.147007 | 0.099112 | 0.090135 | 0.120053 | 0.590855 | 0.104423 | 1.155703 |
| 1:1 | Average ct | 20.78167 | 34.7692 | 22.60394 | 18.02783 | 19.0593 | 18.98917 | 18.83221 | 23.34986 | 22.11225 | 27.94057 | 21.57148 | 32.44552 |
|  | std | 0.184829 | 0 | 0.134872 | 0.253758 | 0.296477 | 0.127719 | 0.182201 | 0.018824 | 0.374701 | 0.843942 | 0.381675 | 0.397824 |
| 1:3 | Average ct | 20.34388 | 31.90403 | 21.02166 | 18.01583 | 18.21057 | 19.07747 | 17.95534 | 22.08023 | 20.96788 | 27.97895 | 21.77259 | 32.94818 |
|  | std | 0.523434 | 0 | 0.262559 | 0.051113 | 0.199302 | 0.168584 | 0.078631 | 0.274947 | 0.169037 | 0.593512 | 0.353192 | 0.815643 |
| MC | Average ct | 19.3689 | 32.99489 | 20.50285 | 17.37358 | 17.35392 | 18.39408 | 17.45627 | 21.25777 | 19.84663 | 25.52718 | 20.80653 | 30.91438 |
|  | std | 0.596324 | 1.869737 | 0.312886 | 0.308079 | 0.459624 | 0.427196 | 0.639002 | 0.322304 | 0.593544 | 0.343722 | 0.226546 | 0.401497 |
|  |  | Actb | B2m | Hprt1 | Ldha | Rplp1 | RGDC | RTC | RTC | RTC | PPC | PPC | PPC |
| 3:1 | Average ct | 16.47359 | 18.29221 | 21.61194 | 20.66157 | 18.0764 | 0 | 0 | 0 | 0 | 18.55303 | 18.57169 | 18.88839 |
|  | std | 0.134827 | 0.07855 | 0.086894 | 0.08835 | 0.480305 | 0 | 0 | 0 | 0 | 0.038082 | 0.067481 | 0.247274 |
| 1:1 | Average ct | 16.87256 | 18.58381 | 21.51182 | 20.76671 | 18.31151 | 0 | 0 | 0 | 0 | 18.485 | 18.43447 | 18.56869 |
|  | std | 0.384662 | 0.115918 | 0.621976 | 0.05551 | 0.524243 | 0 | 0 | 0 | 0 | 0.397744 | 0.293991 | 0.248741 |
| 1:3 | Average ct | 16.24093 | 18.06725 | 21.02543 | 19.92725 | 16.7909 | 0 | 0 | 0 | 0 | 18.77194 | 18.84069 | 19.29619 |
|  | std | 0.488983 | 0.015272 | 0.154463 | 0.071199 | 0.096465 | 0 | 0 | 0 | 0 | 0.475477 | 0.344137 | 0.69523 |
| MC | Average ct | 15.51155 | 17.67659 | 20.52302 | 19.28877 | 16.47217 | 0 | 0 | 0 | 0 | 18.85897 | 18.76672 | 19.08844 |
|  | std | 0.325246 | 0.345321 | 0.18031 | 0.43139 | 0.228726 | 0 | 0 | 0 | 0 | 0.061279 | 0.076823 | 0.345129 |
